# Supplementary material for: Extrusion of BMP2+ surface colonocytes promotes stromal remodeling and tissue regeneration
Source: Nat Commun. 2025 May 3;16:4131. doi: 10.1038/s41467-025-59474-y (PMC12049494; doi:10.1038/s41467-025-59474-y)
Supplement: Supplementary file 1 — Supplementatary Information [file 41467_2025_59474_MOESM1_ESM.pdf]

Supplementary Information

Extrusion of BMP2+ surface colonocytes promotes stromal remodeling and tissue regeneration

Julian Heuberger, Lichao Liu, Hilmar Berger, Joop van den Heuvel, Manqiang Lin, Stefanie Müllerke, Safak Bayram, Giulia Beccaceci, Hugo de Jonge, Ermanno Gherardi, Michael Sigal

This PDF file includes:

Supplementary Table 1, Supplementary Figures 1-8

Supplementary Table 1

Corresponding P-values:

celltype	GeneSymbol	p_val	avg_log2FC	pct.1	pct.2	p_val_adj
**Colonocytes**	Bmp2	1.063813E-08	-1.0327533	0.277	0.592	0.0002419535
**SC**	Bmp2	1.801412E-17	-1.4318483	0.03	0.15	4.097131E-13
**Secretory**	Bmp2	1.346806E-10	-1.2795962	0.038	0.256	3.063175E-06
**TA**	Bmp2	1.184192E-19	-0.9140337	0.112	0.34	2.693326E-15
**Colonocytes**	Krt20	0.3164751	0.9638114	0.375	0.374	1
**SC**	Krt20	0.8088588	0.6159561	0.02	0.018	1
**Secretory**	Krt20	0.0007944942	-2.5921778	0.023	0.098	1
**TA**	Krt20	0.7998547	1.1080759	0.037	0.035	1

Supplementary Table 1: Corresponding P-values of Bmp2 and Krt20 expression in the scRNA-seq dataset from progenitors with or without IFN-γ treatment, related to Extended Dara Figure 2. The p-values were determined using one-way ANOVA (two-sided) with no adjustments for multiple comparisons.

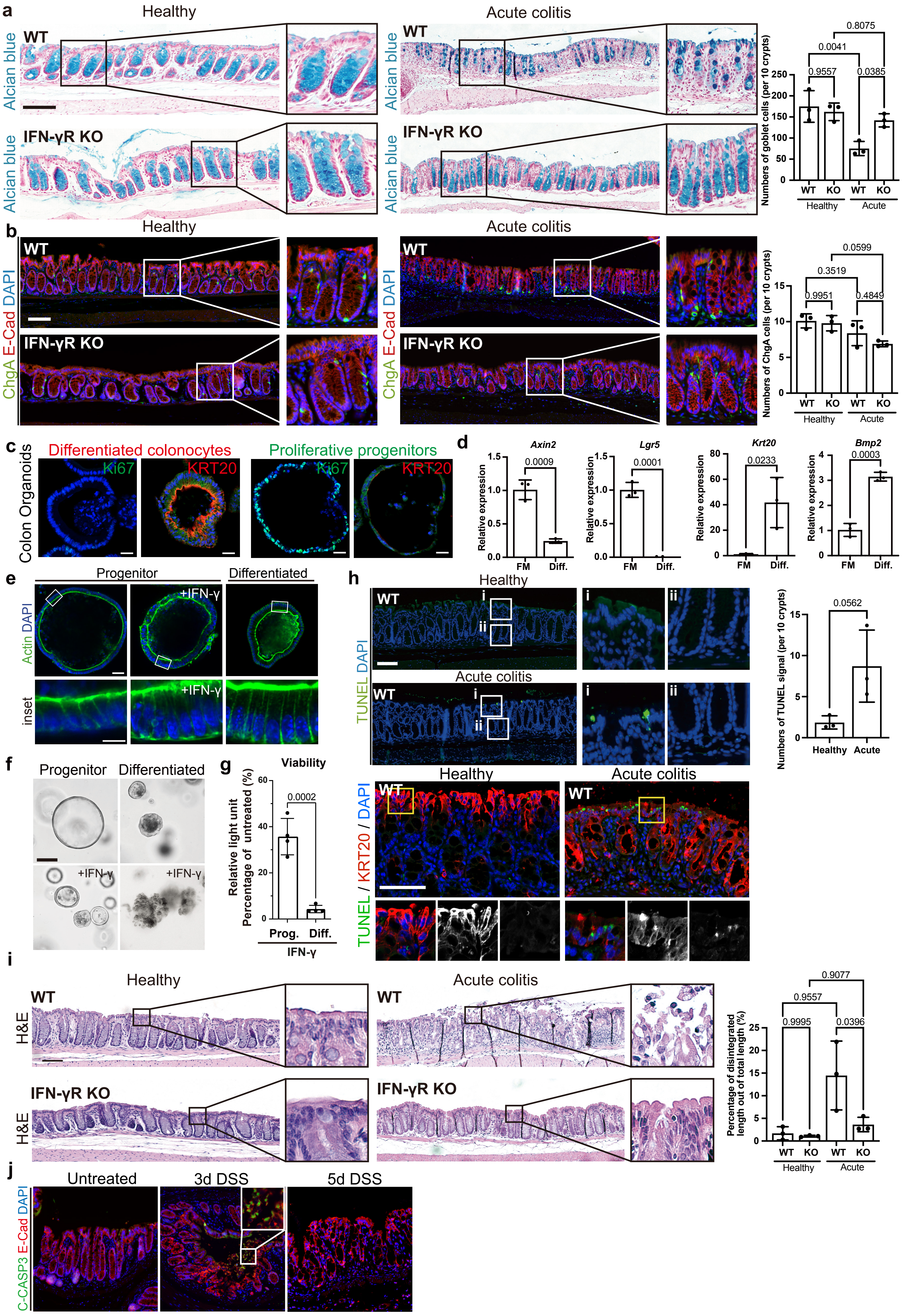
a
Healthy
Acute colitis
Alcian blue
WT
IFN-γR KO
Numbers of goblet cells (per 10 crypts)
0.8075
0.0041
0.9557
0.0385
WT
KO
Healthy
Acute
b
ChgA E-Cad DAPI
Numbers of ChgA cells (per 10 crypts)
0.0599
0.3519
0.9951
0.4849
c
Colon Organoids
Differentiated colonocytes
Proliferative progenitors
Ki67
KRT20
d
Axin2
Lgr5
Krt20
Bmp2
Relative expression
0.0009
0.0001
0.0233
0.0003
FM
Diff.
e
Progenitor
Differentiated
+IFN-γ
Actin DAPI
inset
f
Progenitor
Differentiated
+IFN-γ
g
Viability
Relative light unit
Percentage of untreated (%)
0.0002
Prog.
Diff.
IFN-γ
h
Healthy
Acute colitis
TUNEL DAPI
Numbers of TUNEL signal (per 10 crypts)
0.0562
Healthy
Acute
TUNEL / KRT20 / DAPI
i
H&E
Percentage of disintegrated length out of total length (%)
0.9077
0.9557
0.9995
0.0396
j
Untreated
3d DSS
5d DSS
C-CASP3 E-Cad DAPI

Supplementary Fig.1: IFN-γ-induced loss of goblet cells and colonocyte death, related to Figure 1. (a) Alcian blue staining (×200 magnification) showing goblet cells in acute colitis mice compared to untreated controls, as well as in WT and IFN-γR KO mice, quantification on the right. Scale bar: 100 µm. n=3 mice. **(b)** Neuroendocrine cells were analyzed by IF staining (×200 magnification) of chromogranin A (ChgA) in acute colitis and healthy WT and IFN-γR KO mice, quantification on the right. Scale bar: 100 µm. n=3 mice. **(c)** IF staining (×600 magnification) of Ki67 and KRT20 was performed to distinguish between proliferative progenitors and differentiated colonocytes. Scale bars: 30 µm. **(d)** qPCR was used to compare the expression of *Axin2*, *Lgr5*, *Krt20*, and *Bmp2* between proliferative progenitors (FM) and differentiated colonocytes (Diff.). n=3 mice. **(e)** Actin/phalloidin staining (×600 magnification) was performed to indicate cell polarity in progenitors with or without IFN-γ treatment and differentiated colonocytes. Scale bars: 30 µm. **(f)** Progenitor organoids and differentiated organoids were treated with 50 ng/ml IFN-γ for 48 h. Scale bar: 100 µm. (×200 magnification) **(g)** Progenitors (Prog.) and differentiated organoids were treated with IFN-γ for 20 h, and apoptotic cells were detected using the CellTiter-Glo® Luminescent Cell Viability Assay. n=4 mice. **(h)** Apoptotic cells detected by TUNEL staining (×200 magnification) in healthy and acute colitis tissue of WT mice, with quantification on the right. Co-staining of TUNEL and KRT20 (×600 magnification) was shown on the lower panel. Scale bar: 100 µm. n=3 mice. **(i)** H&E staining (×200 magnification) indicating the disintegrated area in healthy and acute colitis colon tissue of WT and IFN-γR KO mice; quantification on the right. Scale bar: 100 µm. n=3 mice. **(j)** C-CASP3 staining (×200 magnification) was performed at different time points (3 and 5 days DSS) of DSS colitis samples. Scale bar: 100 µm. Data are represented as mean ± SD. The p-values in (a), (b), and (i) were determined using one-way ANOVA (two-sided) with no adjustments for multiple comparisons. The p-values in (d), (g), and (h) were determined using unpaired Student's t-tests (two-sided).

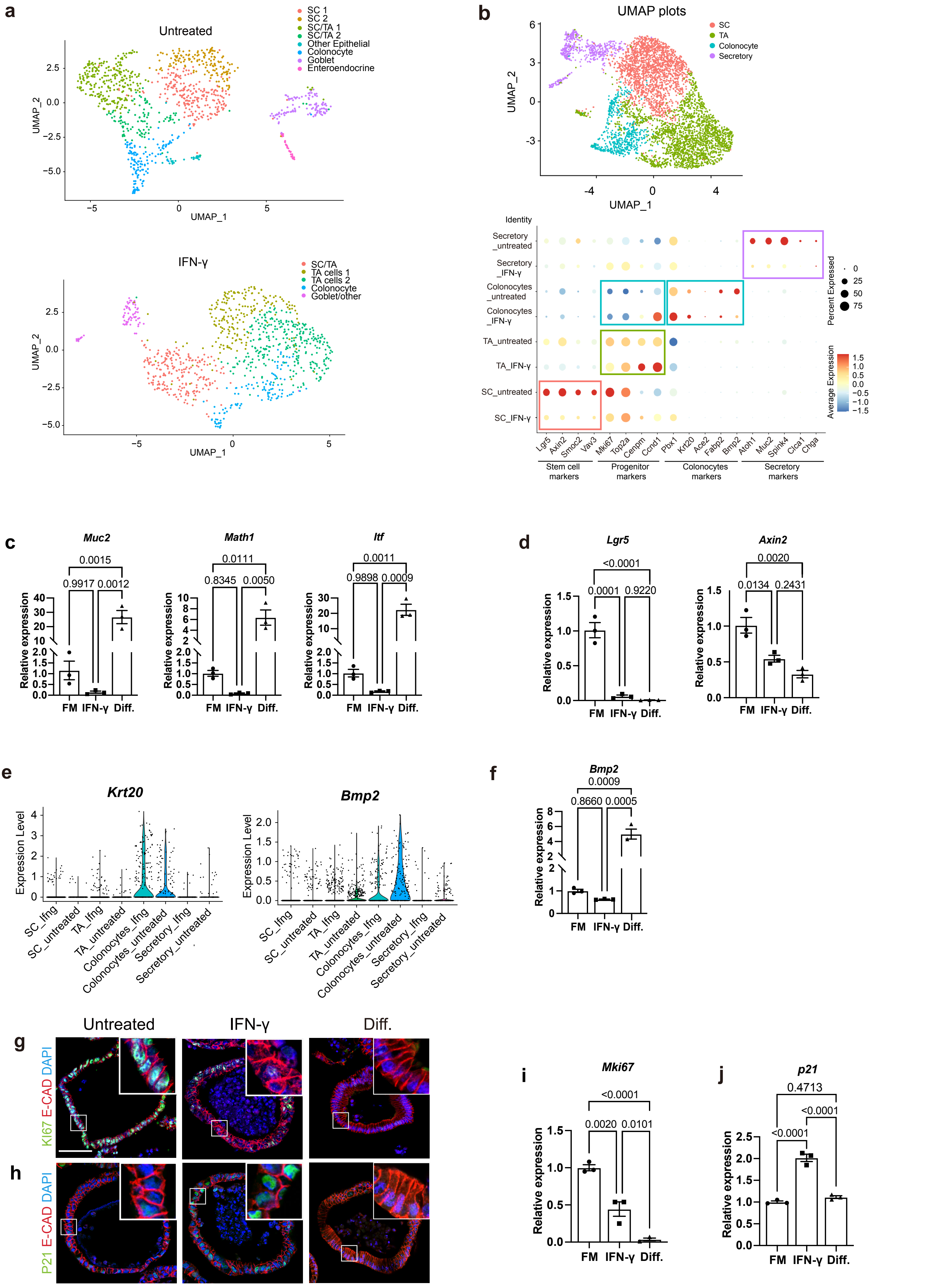
a
Untreated
SC 1
SC 2
SC/TA 1
SC/TA 2
Other Epithelial
Colonocyte
Goblet
Enteroendocrine
UMAP_2
UMAP_1
IFN-γ
SC/TA
TA cells 1
TA cells 2
Colonocyte
Goblet/other
b
UMAP plots
SC
TA
Colonocyte
Secretory
Identity
Secretory_untreated
Secretory_IFN-γ
Colonocytes_untreated
Colonocytes_IFN-γ
TA_untreated
TA_IFN-γ
SC_untreated
SC_IFN-γ
Lgr5 Axin2 Smoc2 Vav3 Mki67 Top2a Cenpm Ccnd1 Pbx1 Krt20 Ace2 Fabp2 Bmp2 Atoh1 Muc2 Spink4 Clca1 Chga
Stem cell markers
Progenitor markers
Colonocytes markers
Secretory markers
Percent Expressed
Average Expression
c
Muc2
Math1
Itf
Relative expression
FM IFN-γ Diff.
0.0015 0.9917 0.0012
0.0111 0.8345 0.0050
0.0011 0.9898 0.0009
d
Lgr5
Axin2
<0.0001 0.0001 0.9220
0.0020 0.0134 0.2431
e
Krt20
Bmp2
Expression Level
SC_Ifng
SC_untreated
TA_Ifng
TA_untreated
Colonocytes_Ifng
Colonocytes_untreated
Secretory_Ifng
Secretory_untreated
f
Bmp2
0.0009 0.8660 0.0005
g
h
Untreated
IFN-γ
Diff.
KI67 E-CAD DAPI
P21 E-CAD DAPI
i
Mki67
<0.0001 0.0020 0.0101
j
p21
0.4713 <0.0001 <0.0001

Supplementary Fig.2: IFN-γ-induced colonocytes are distinct from homeostatic colonocytes

(a) UMAP plot illustrating different cell clusters in sc-RNAseq dataset from untreated progenitors (upper) and IFN-γ treated progenitors (lower). SC: stem cells. TA: Transit-amplifying cells. **(b)** UMAP and Dot plot illustrating the sc-RNAseq dataset from progenitors with or without IFN-γ treatment as well as the expression of known marker genes against detected epithelial cell clusters in FM organoids identified by sc-RNAseq. Circle size represents the within-cluster probability of gene detection, while fill color represents the normalized average expression level. **(c)** mRNA expression of goblet cell markers (*Muc2*, *Math1*, and *Itf*) as assessed by RT-PCR in organoids grown in FM with, without IFN-γ treatment, or in differentiation medium; n = 3 mice. (**d**) qPCR comparing progenitor markers *Axin2* and *Lgr5.* **(e)** Violin plots showing the expression levels of *Krt20* and *Bmp2* in progenitor cells, comparing untreated and IFN-γ-treated conditions. (**f**), mRNA expression of the differentiation marker *Bmp2* assessed by RT-PCR in organoids grown in FM with or without IFN-γ treatment or in differentiation medium; n = 3 mice. (**g, h**) IF staining (×600 magnification) illustrating the differential expression Ki67 (proliferation marker) and p21 (cell cycle arrest target) in progenitors, IFN-γ-treated progenitors, and differentiated colonocytes. Scale bars: 40 µm. **(i)** RT-PCR for the proliferation marker *mKi67* in the indicated groups as in **(c);** n = 3 mice per group. **(j)** RT-PCR for the cell cycle arrest marker *p21* in the indicated groups as in **(c);** n = 3 mice per group. (c, d, g, j and k) p-values were determined using one-way ANOVA (two-sided) with no adjustments for multiple comparisons. (All data are represented as mean ± SD).

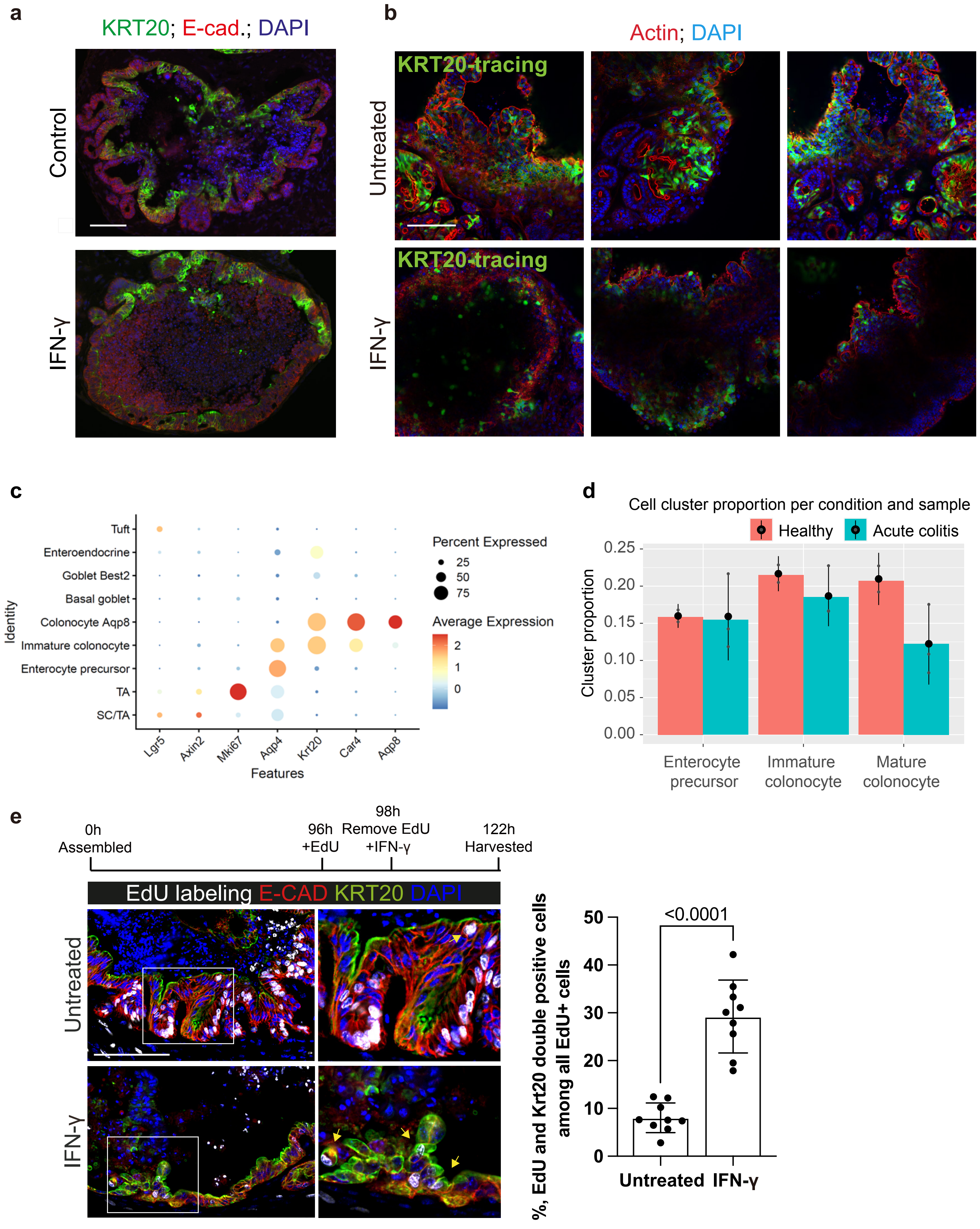
a
KRT20; E-cad.; DAPI
Control
IFN-γ
b
Actin; DAPI
KRT20-tracing
Untreated
KRT20-tracing
IFN-γ
c
Tuft
Enteroendocrine
Goblet Best2
Basal goblet
Colonocyte Aqp8
Immature colonocyte
Enterocyte precursor
TA
SC/TA
Identity
Lgr5
Axin2
Mki67
Aqp4
Krt20
Car4
Aqp8
Features
Percent Expressed
25
50
75
Average Expression
2
1
0
d
Cell cluster proportion per condition and sample
Healthy
Acute colitis
Cluster proportion
0.25
0.20
0.15
0.10
0.05
0.00
Enterocyte precursor
Immature colonocyte
Mature colonocyte
e
0h
Assembled
96h
+EdU
98h
Remove EdU
+IFN-γ
122h
Harvested
EdU labeling E-CAD KRT20 DAPI
Untreated
IFN-γ
%, EdU and Krt20 double positive cells among all EdU+ cells
50
40
30
20
10
0
<0.0001
Untreated
IFN-γ

Supplementary Fig.3: IFN-γ decreases the proportion of mature colonocytes in vivo and accelerates the differentiation of progenitors in assembloids, related to Figure 2. (a) Overview images (×200 magnification) of assembloids stained for KRT20 and E-cadherin in the untreated state (left) and after treatment with IFN-γ (right). Scale bar: 100 µm. **(b)** Additional images (×600 magnification) of whole-mount staining of assembloids co-stained with actin/phalloidin derived from *Krt20CreERT2/Rosa26-tdTomato* mice. Krt20 was traced using 800 nM tamoxifen to label the differentiated lineages. Scale bar: 100 µm. **(c)** Dot blot illustrating the definition of cell populations based on their marker gene expression. SC: stem cells. TA: Transit-amplifying cells. **(d)** The proportion of enterocyte precursors, immature and mature colonocytes in mice with 6 days of DSS treatment (acute colitis) and their associated controls (healthy), as determined by sc-RNAseq from an online dataset (GSE201723), presented as absolute numbers. Data are represented as mean +/- 2 SEM. n=2 control mice, n=3 DSS mice. (**e**) Proliferative cells were labeled with EdU and co-stained with KRT20 and E-cadherin. Images were taken via Observer 7 with ×200 magnification. The scheme of EdU labeling is presented in the upper left panel and quantifications are shown in the right panel. n=9 individual assembloids from 3 biological replicates. Scale bar: 100 µm. The p-value was determined using Student's t-test (two-sided). Data are represented as mean ± SD.

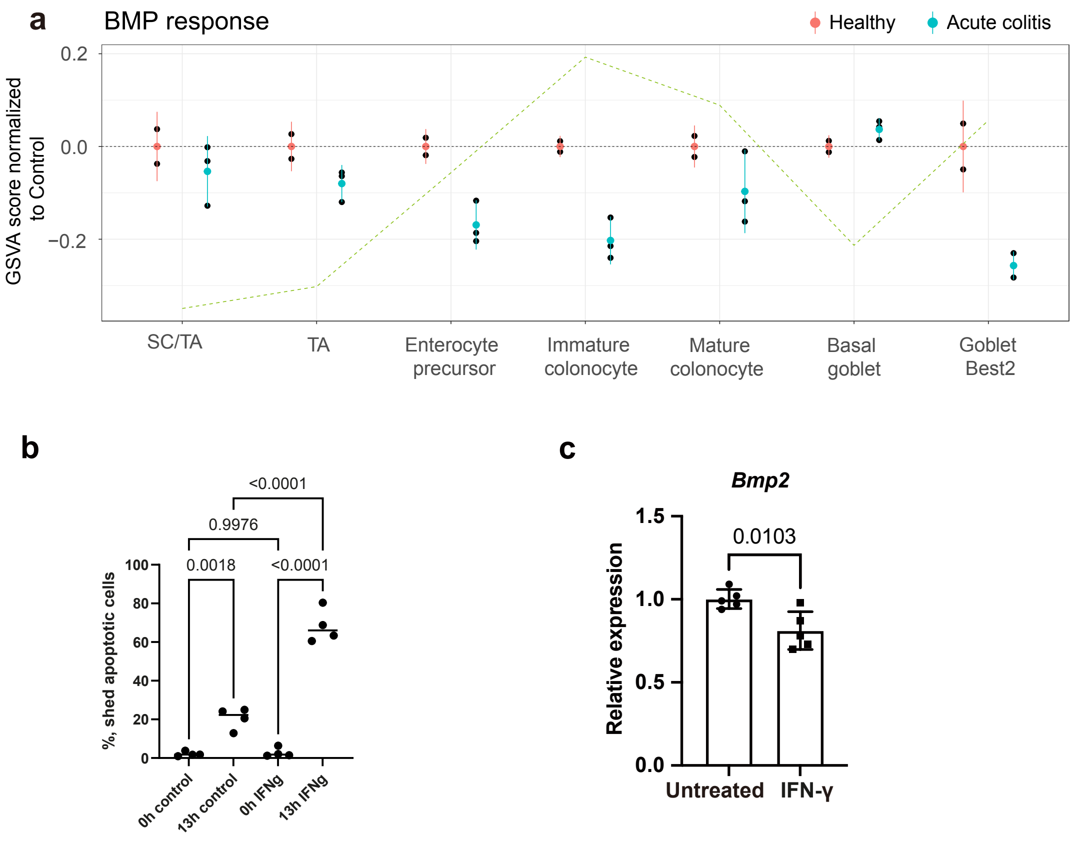
a
BMP response
Healthy
Acute colitis
GSVA score normalized to Control
0.2
0.0
−0.2
SC/TA
TA
Enterocyte precursor
Immature colonocyte
Mature colonocyte
Basal goblet
Goblet Best2
b
%, shed apoptotic cells
100
80
60
40
20
0
<0.0001
0.9976
0.0018
<0.0001
0h control
13h control
0h IFNg
13h IFNg
c
Bmp2
Relative expression
1.5
1.0
0.5
0.0
0.0103
Untreated
IFN-γ

Supplementary Fig.4: BMP signaling is inhibited in colitis and IFN-γ decreases BMP2 expression in assembloids, related to Figure 4. (a) BMP2 coexpressed gene signature activity by cell population upon DSS (acute colitis) *in vivo,* as measured by GSVA enrichment score above the average level in control (healthy) mice from an online sc-RNAseq dataset (GSE201723). Dashed line indicates average control level for each population. SC: stem cells. TA: Transit-amplifying cells. n=3 mice per group. Data are represented as mean ± 2 SEM. **(b)** Quantification of apoptotic and shed cells of control and IFN-γ treated organoids, p-values were determined using one-way ANOVA (two-sided) with no adjustments for multiple comparisons. n=4 mice per group. **(c)** qRT-PCR of *Bmp2* in assembloids with or without IFN-γ treatment. n=5 mice. The p-value was determined by Student's t-test (two-sided). Data are represented as mean ± SD.

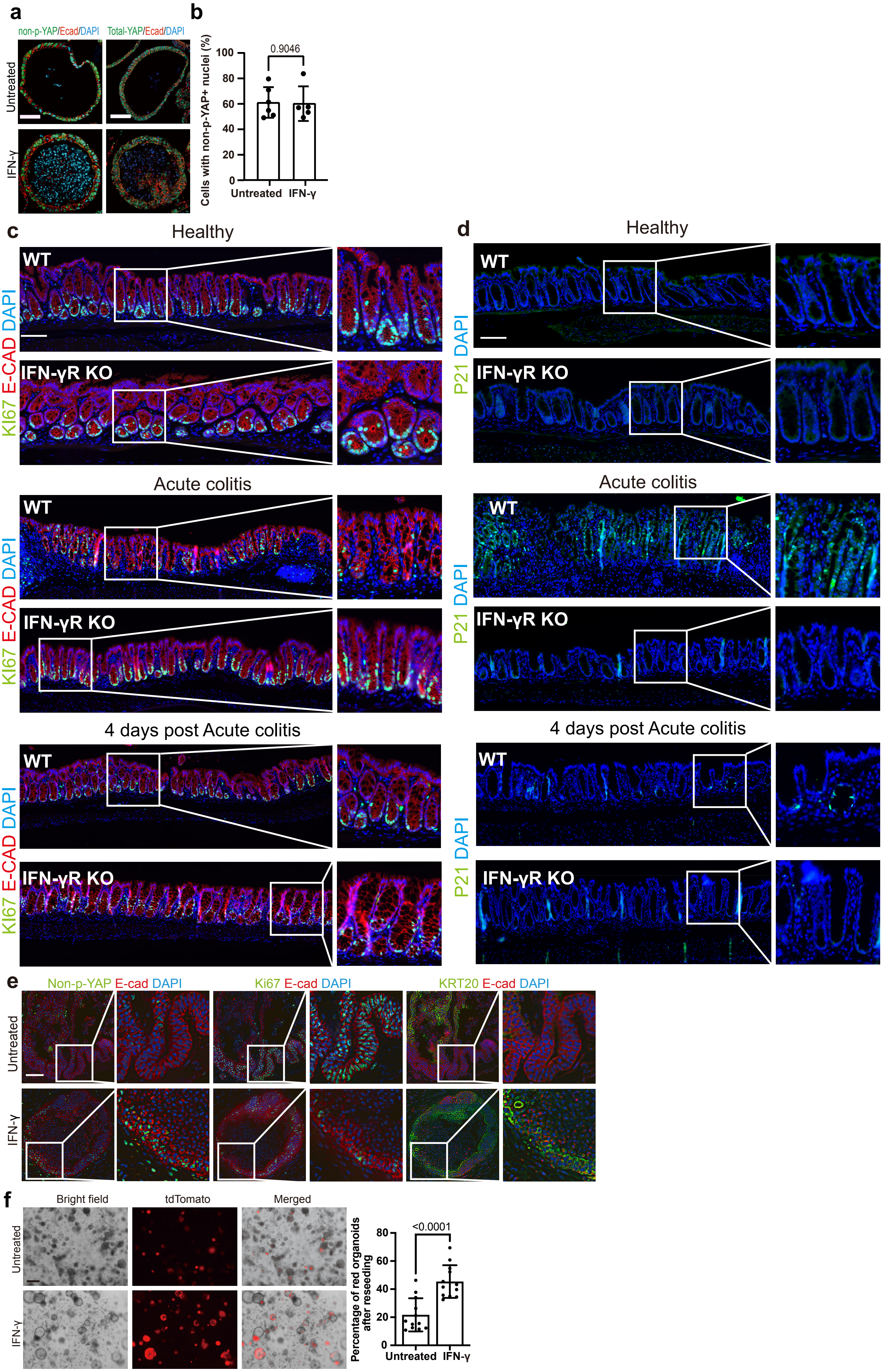
a
non-p-YAP/Ecad/DAPI
Total-YAP/Ecad/DAPI
Untreated
IFN-γ
b
0.9046
Cells with non-p-YAP+ nuclei (%)
100
80
60
40
20
0
Untreated
IFN-γ
c
Healthy
WT
IFN-γR KO
KI67 E-CAD DAPI
Acute colitis
WT
IFN-γR KO
KI67 E-CAD DAPI
4 days post Acute colitis
WT
IFN-γR KO
KI67 E-CAD DAPI
d
Healthy
WT
IFN-γR KO
P21 DAPI
Acute colitis
WT
IFN-γR KO
P21 DAPI
4 days post Acute colitis
WT
IFN-γR KO
P21 DAPI
e
Non-p-YAP E-cad DAPI
Ki67 E-cad DAPI
KRT20 E-cad DAPI
Untreated
IFN-γ
f
Bright field
tdTomato
Merged
Untreated
IFN-γ
<0.0001
Percentage of red organoids after reseeding
80
60
40
20
0
Untreated
IFN-γ

Supplementary Fig.5: IFN-γ inhibits colonic epithelial proliferation and induces cell cycle arrest, related to Figure 5. (a) IF staining (×600 magnification) of non-phosphorylated YAP (non-p-YAP) and total YAP was performed in progenitor organoids with IFN-γ treatment and non-p-YAP was quantified **(b)**; Scale bar: 50 µm; Control: n=6 mice; IFN-γ-treated: n=5 mice. **(c)** Proliferative activity was assessed by Ki67 via IF staining (×200 magnification) in healthy control, acute colitis, and repair phase of WT and IFN-γR KO mice. Scale bar: 100 µm. **(d)** Cell cycle arrest was evaluated by p21 staining via IF (×200 magnification) in healthy control, acute colitis, and repair phase of WT and IFN-γR KO mice. Scale bar: 100 µm. **(e)** The effect of IFN-γ on non-p-YAP, KI67, and KRT20 was visualized using multiplex IF staining (×200 magnification) in assembloids. Scale bar: 100 µm. **(f)** Regrowth of organoids (×200 magnification) in FM derived from KRT20-traced assembloids, either untreated or treated with IFN-γ, originating from *Krt20CreERT2/Rosa26-tdTomato* mice, accompanied by quantification of the percentage of KRT20-traced organoids. n=3 mice. Scale bar: 100 µm. All p-values were determined by Student's t-test (two side). Data are represented as mean ± SD.

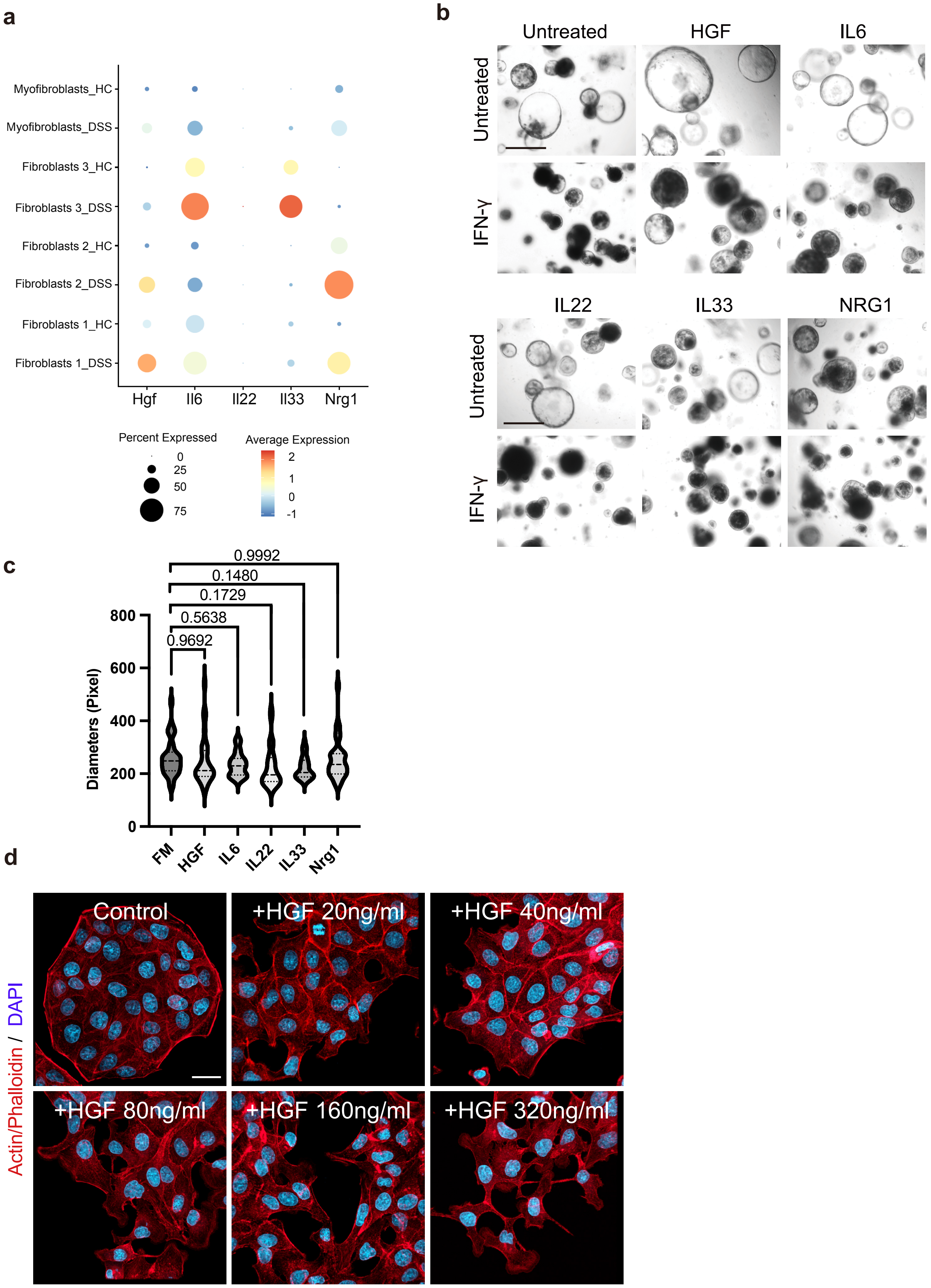

a
Myofibroblasts_HC
Myofibroblasts_DSS
Fibroblasts 3_HC
Fibroblasts 3_DSS
Fibroblasts 2_HC
Fibroblasts 2_DSS
Fibroblasts 1_HC
Fibroblasts 1_DSS
Hgf
Il6
Il22
Il33
Nrg1
Percent Expressed
0
25
50
75
Average Expression
2
1
0
-1
b
Untreated
HGF
IL6
Untreated
IFN-γ
IL22
IL33
NRG1
Untreated
IFN-γ
c
0.9992
0.1480
0.1729
0.5638
0.9692
800
600
400
200
0
Diameters (Pixel)
FM
HGF
IL6
IL22
IL33
Nrg1
d
Control
+HGF 20ng/ml
+HGF 40ng/ml
+HGF 80ng/ml
+HGF 160ng/ml
+HGF 320ng/ml
Actin/Phalloidin / DAPI

Supplementary Fig.6: Growth factors (HGF, IL-6, IL-22, IL-33, and Nrg1) are overexpressed in DSS-induced colitis but do not much affect the growth of colonic organoids in FM, related to Figure 6. **(a)** Single-cell RNA sequencing analysis of alterations in the expression of healing factors in stromal cells in healthy and colitis conditions obtained from an online data set (GSE114374). **(b)** Images (×200 magnification) of progenitor organoids treated with various growth factors (HGF, IL-6, IL-22, IL-33, and NRG1) in FM, and quantifications of organoids diameters were shown in **(c)**. n=30 organoids from 3 biological replicates. Scale bar: 1 mm. The p-values were determined using one-way ANOVA (two-sided) with no adjustments for multiple comparisons. **(d)** MDCK-scatter assay (×600 magnification) to validate the activity of self-produced HGF used at the indicated concentrations for 24 h. MDCK stained with actin-phalloidin and DAPI; scale bar: 25 µm. Data are represented as mean ± SD.

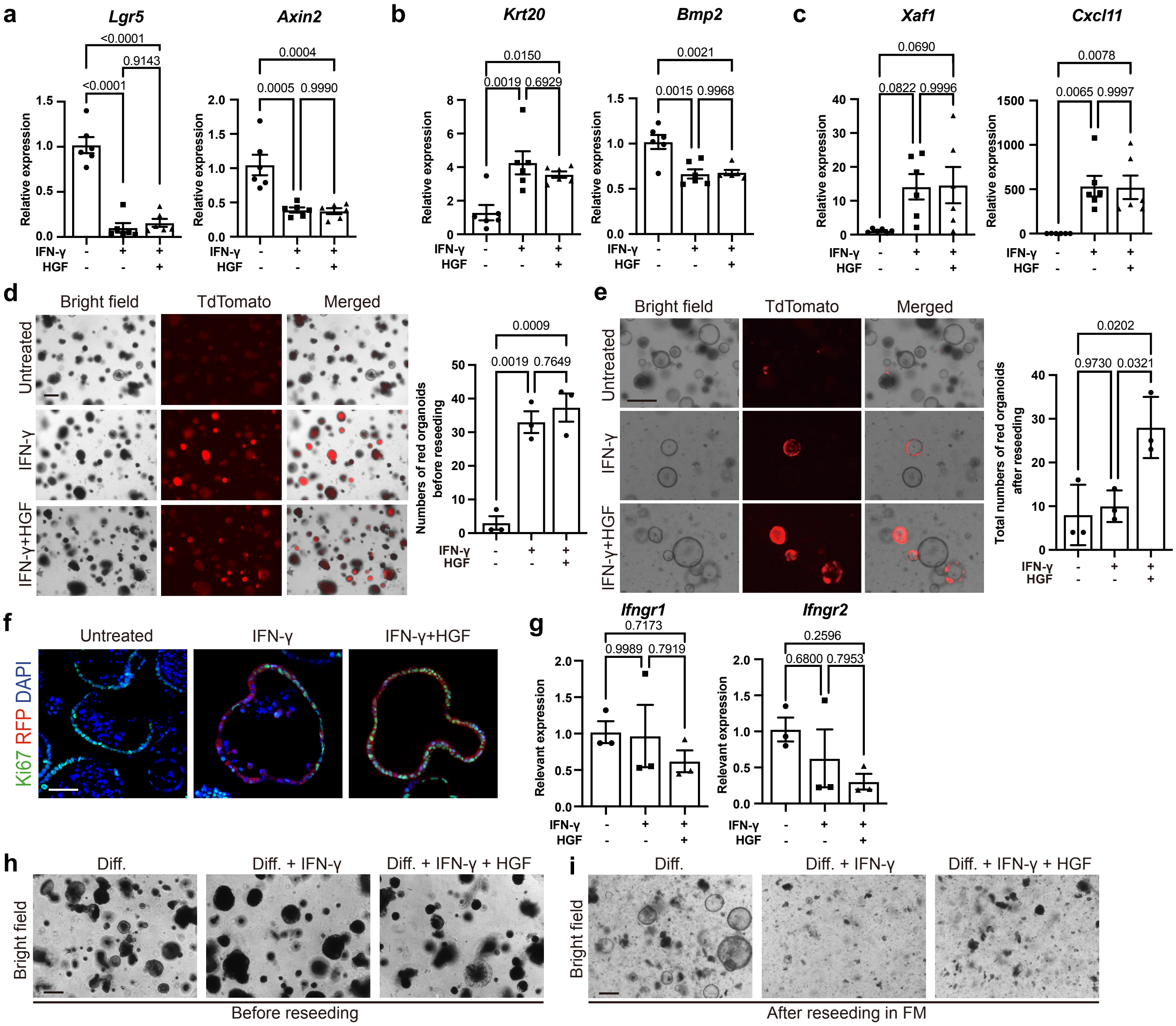
a
Lgr5
Axin2
Relative expression
IFN-γ
HGF
b
Krt20
Bmp2
c
Xaf1
Cxcl11
d
Bright field
TdTomato
Merged
Untreated
IFN-γ
IFN-γ+HGF
Numbers of red organoids before reseeding
e
Total numbers of red organoids after reseeding
f
Untreated
IFN-γ
IFN-γ+HGF
Ki67 RFP DAPI
g
Ifngr1
Ifngr2
Relevant expression
h
Diff.
Diff. + IFN-γ
Diff. + IFN-γ + HGF
Bright field
Before reseeding
i
After reseeding in FM

Supplementary Fig.7: Evaluation of the combined effect of HGF and IFN-γ treatment on progenitor and differentiated colonic cells, related to Figure 7. (a) qPCR for expression of the stem cell marker *Lgr5* and the WNT-target gene *Axin2* in progenitors treated with IFN-γ or IFN-γ + HGF. n=3 mice. **(b)** qPCR for expression of *Krt20* and *Bmp2* in progenitors treated with IFN-γ or IFN-γ + HGF. n=6 mice. **(c)** qPCR for expression of the IFN-γ targets *Xaf1* and *Cxcl11* in progenitors treated with IFN-γ or IFN-γ + HGF. n=6 mice. **(d)** Images of KRT20 tracing in organoids derived from *Krt20CreERT2/Rosa26-tdTomato* mice treated with IFN-γ or IFN-γ + HGF together with 800 nM 4OHT-tamoxifen for 48 h, with the number of traced organoids quantified. Scale bar: 100 µm. n=3 mice. **(e)** Organoids from **(d)** were reseeded into FM. Images of regrowing organoids (×200 magnification) are shown and numbers of KRT20-traced organoids quantified. Scale bar: 100 µm. n=3 mice. **(f)** Co-staining of KI67 and RFP (×200 magnification) in organoids harvested from **(e)** to evaluate proliferation of KRT20-traced cells. Scale bar: 100 µm. **(g)** qPCR for expression of IFN-γ receptors, *Ifngr1* and *Ifngr2*, in progenitors treated with IFN-γ or IFN-γ + HGF. n=3 mice. **(h)** Brightfield images (×200 magnification) of differentiated organoids (Diff.) with IFN-γ or IFN-γ + HGF treatment for 48 h, followed by reseeding into FM **(i)**; Scale bar: 100 µm. All p-values were determined using one-way ANOVA (two-sided) with no adjustments for multiple comparisons. Data are represented as mean ± SD.

a

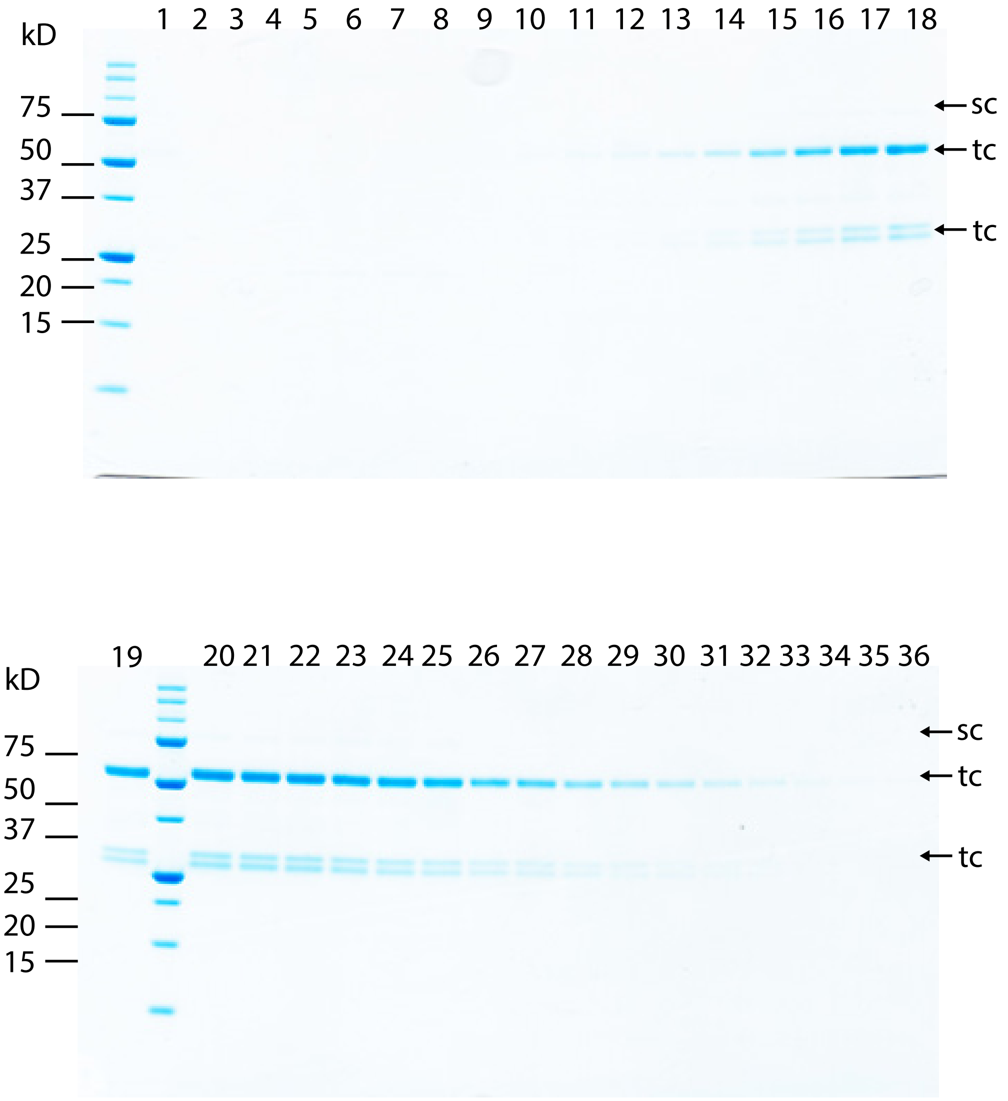
1 2 3 4 5 6 7 8 9 10 11 12 13 14 15 16 17 18
kD
75
50
37
25
20
15
sc
tc
tc
19 20 21 22 23 24 25 26 27 28 29 30 31 32 33 34 35 36
kD
75
50
37
25
20
15
sc
tc
tc

Supplementary Fig.8: Purification of HGF produced in CHO Lec3.2.8.1 cells. (a) Elution from HGF/SF from MonoS GL column Sample pooled were F17 to F36 range from 620 mM to 770 mM NaCl, demonstrating high purity of produced tc-HGF.
