# Supplementary material for: Extrusion of BMP2+ surface colonocytes promotes stromal remodeling and tissue regeneration
Source: Nat Commun. 2025 May 3;16:4131. doi: 10.1038/s41467-025-59474-y (PMC12049494; doi:10.1038/s41467-025-59474-y)
Supplement: Supplementary file 2 — Description Of Additional Supplementary File [file 41467_2025_59474_MOESM2_ESM.pdf]

Description of Additional supplementary files

Supplementary Movie 1: Live-cell imaging of progenitor cells and differentiated colonocytes with or without IFN-γ treatment for 23 h, related to Figure 1. Scale bar: 100 µm.

Supplementary Movie 2: Live imaging of progenitor cells in untreated, IFN-γ treated, and IFN-γ + HGF treated conditions for 24 h, related to Figure 6. Scale bar: 30 µm.

Supplementary Movie 3: Live imaging of progenitor cells in untreated and IFN-γ treated conditions for 13 h with apoptotic cell staining. Scale bar: 50 µm. Quantification see Extended Data Fig. 4b

Supplementary Data1: Regulated genes in Krt20+ colonocytes treated with IFN-γ compared to untreated control using sc-RNAseq
