# Supplementary material for: Extrusion of BMP2+ surface colonocytes promotes stromal remodeling and tissue regeneration
Source: Nat Commun. 2025 May 3;16:4131. doi: 10.1038/s41467-025-59474-y (PMC12049494; doi:10.1038/s41467-025-59474-y)
Supplement: Supplementary file 7 — Reporting summary [file 41467_2025_59474_MOESM7_ESM.pdf]

Corresponding author(s): Michael Sigal
Last updated by author(s): Mar 25, 2025

Reporting Summary

Nature Portfolio wishes to improve the reproducibility of the work that we publish. This form provides structure for consistency and transparency in reporting. For further information on Nature Portfolio policies, see our Editorial Policies and the Editorial Policy Checklist.

Statistics

For all statistical analyses, confirm that the following items are present in the figure legend, table legend, main text, or Methods section.

n/a	Confirmed	
☐	☒	The exact sample size (*n*) for each experimental group/condition, given as a discrete number and unit of measurement
☐	☒	A statement on whether measurements were taken from distinct samples or whether the same sample was measured repeatedly
☐	☒	The statistical test(s) used AND whether they are one- or two-sided<br>*Only common tests should be described solely by name; describe more complex techniques in the Methods section.*
☒	☐	A description of all covariates tested
☐	☒	A description of any assumptions or corrections, such as tests of normality and adjustment for multiple comparisons
☐	☒	A full description of the statistical parameters including central tendency (e.g. means) or other basic estimates (e.g. regression coefficient) AND variation (e.g. standard deviation) or associated estimates of uncertainty (e.g. confidence intervals)
☐	☒	For null hypothesis testing, the test statistic (e.g. F, t, r) with confidence intervals, effect sizes, degrees of freedom and P value noted<br>*Give P values as exact values whenever suitable.*
☒	☐	For Bayesian analysis, information on the choice of priors and Markov chain Monte Carlo settings
☒	☐	For hierarchical and complex designs, identification of the appropriate level for tests and full reporting of outcomes
☒	☐	Estimates of effect sizes (e.g. Cohen's *d*, Pearson's *r*), indicating how they were calculated

Our web collection on statistics for biologists contains articles on many of the points above.

Software and code

Policy information about availability of computer code

Data collection	QuantStudio 3 Real-Time PCR Software vl.7.1 (Thermo Fisher)<br>ZEN 3.4<br>Adobe Illustrator 26.3.1
Data analysis	ImageJ 2.0.0<br>GraphPad Prism 8<br>RStudio 2021.09.1<br>R4.2.2<br>R packages (Seurat v3, GSVA 1.48.0, pheatmap 1.0.12, Harmony 0.1.153)

For manuscripts utilizing custom algorithms or software that are central to the research but not yet described in published literature, software must be made available to editors and reviewers. We strongly encourage code deposition in a community repository (e.g. GitHub). See the Nature Portfolio guidelines for submitting code & software for further information.

Data

Policy information about availability of data

All manuscripts must include a data availability statement. This statement should provide the following information, where applicable:

- Accession codes, unique identifiers, or web links for publicly available datasets
- A description of any restrictions on data availability
- For clinical datasets or third party data, please ensure that the statement adheres to our policy

All scRNAseq data generated in this study have been deposited in the National Centre for Biotechnology, Gene Expression Information Omnibus (GEO) under accession code GSE255366 [https://www.ncbi.nlm.nih.gov/geo/query/acc.cgi?acc=GSE255366]. The previously published scRNAseq data are available in the GEO under accession code GSE201723 [https://www.ncbi.nlm.nih.gov/geo/query/acc.cgi?acc=GSE201723] and GSE114374 [https://www.ncbi.nlm.nih.gov/geo/query/acc.cgi?acc=GSE114374]. Source data are provided if required.

Research involving human participants, their data, or biological material

Policy information about studies with human participants or human data. See also policy information about sex, gender (identity/presentation), and sexual orientation and race, ethnicity and racism.

Reporting on sex and gender	No human data included.
Reporting on race, ethnicity, or other socially relevant groupings	No human data included.
Population characteristics	No human data included.
Recruitment	No human data included.
Ethics oversight	No human data included.

Note that full information on the approval of the study protocol must also be provided in the manuscript.

Field-specific reporting

Please select the one below that is the best fit for your research. If you are not sure, read the appropriate sections before making your selection.

☒ Life sciences ☐ Behavioural & social sciences ☐ Ecological, evolutionary & environmental sciences

For a reference copy of the document with all sections, see nature.com/documents/nr-reporting-summary-flat.pdf

Life sciences study design

All studies must disclose on these points even when the disclosure is negative.

Sample size	No statistical methods were used to predetermine sample size. All experiments were performed with n=3 biological replicates except where stated otherwise. Sample sizes were chosen based on available resources and community standards
Data exclusions	No data were excluded for the analysis
Replication	All attempts at replication were successful. All graphs represent data with at least three biological replicates. All images represent findings reproduced at least two times in the laboratory
Randomization	The mice and assembloids/organoids/stromal cells were randomized for respective treatments
Blinding	Group allocation and analysis were done in a blinded fashion.

Reporting for specific materials, systems and methods

We require information from authors about some types of materials, experimental systems and methods used in many studies. Here, indicate whether each material, system or method listed is relevant to your study. If you are not sure if a list item applies to your research, read the appropriate section before selecting a response.

Materials & experimental systems

n/a	Involved in the study
☐	☒ Antibodies
☒	☐ Eukaryotic cell lines
☒	☐ Palaeontology and archaeology
☐	☒ Animals and other organisms
☒	☐ Clinical data
☒	☐ Dual use research of concern
☒	☐ Plants

Methods

n/a	Involved in the study
☒	☐ ChIP-seq
☒	☐ Flow cytometry
☒	☐ MRI-based neuroimaging

Antibodies

Antibodies used

Rabbit-anti-Keratin 20 (Cell Signaling Technology, 13063S, colone D9Z1Z, 1:100);
rabbit anti-KI67 (Cell Signaling Technology, 9129S, clone D3B5, 1:300);
rabbit anti-acitve YAP1 (Abcam, ab205270, clone EPR19812, 1:200);
rabbit anti-p21 (Abcam, ab188224, clone EPR18021, 1:300)
rabbit anti-YAP (Cell Signaling Technology, 14074S, clone D8H1X, 1:200);
rabbit anti-Cleaved Caspase3 (Cell Signaling Technology, 9661S, clone Asp175, 1:200);
rabbit anti-chromogranin A (Abcam, ab283265, RM1025,1:300)
mouse anti-E-cadherin (BD Biosciences, 610181, clone 36/E-Cadherin, 1:300);
Goat anti-RFP (Rockland, 200-101-379S, clone 234aa, 1:200);
Alexa Fluor 647-conjugated phalloidin (Life Technologies, A22287, 1:100);
Alexa Fluor 488 donkey anti-mouse IgG (Jackson Immunoresearch, 715-546-150, 1:300);
Cy3 donkey anti-rabbit IgG (Jackson Immunoresearch, 711-166-152, 1:300);
AlexaFluor 647 donkey anti-goat IgG (Jackson Immunoresearch, 705-605-003, 1:300).

Validation

Validation information of commercial antibodies used in the study:
Rabbit anti-KI67 was used to stain murine proliferative cells. We detected specific staining with the expected pattern. Further validation data can be found under https://www.cellsignal.com/products/primary-antibodies/ki-67-d3b5-rabbit-mab/9129
Rabbit anti-keratin 20 was used to stain murine colonocytes. We detected specific staining with the expected pattern. Further validation data can be found under https://www.cellsignal.com/products/primary-antibodies/keratin-20-d9z1z-xp-rabbit-mab/13063?gclid=CjwKCAjw5ImwBhBtEiwAFHDZx_PVh1K76Ifh-4YwGdqrzJML_l06xFY9Z1958c3Ds3RA-7ifTu-_LhoCjSAQAvD_BwE&gclsrc=aw.ds
Rabbit anti-active YAP1 was used to stain active YAP1 in murine colonic cells. We detected specific staining with the expected pattern. Further validation data can be found under https://www.abcam.com/ products/primary-antibodies/active-yap1-antibodyeprl9812-ab205270.html
Rabbit anti-cleaved caspase 3 was used to stain murine apoptotic cells. We detected specific staining with the expected pattern Further validation data can be found under https://www.cellsignal.com/products/primary-antibodies/cleaved-caspase-3-asp175-antibody/9661
Rabbit anti-p21 was used to stain p21 in murine colonic cells. We detected specific staining with the expected pattern. Further validation data can be found under https://www.abcam.com/en-de/products/primary-antibodies/p21-antibody-epr18021-ab188224
Rabbit anti-chromogranin A was used to stain murine colonic neuroendocrine cells. We detected specific staining with the expected patternFurther validation data can be found under https://www.abcam.com/en-de/products/primary-antibodies/chromogranin-a-antibody-rm1025-ab283265#tab=datasheet
Rabbit anti-YAP was used to stain total YAP in murine colonic cells. We detected specific staining with the expected pattern Further validation data can be found under https://www.cellsignal.com/products/primary-antibodies/yap-d8h1x-xp-rabbit-mab/14074
Mouse anti-E-cadherin was used to stain murine epithelial cells. We detected specific staining with the expected pattern. Further validation data can be found under http://www.bdbiosciences.com/us/applications/research/stem-cell-research/cancer-research/human/purified-mouse-anti-e-cadherin-36e-cadherin/p/610181.
Goat anti-RFP was used to stain KRT20 and Axin2 Lineage tracing cells. We detected specific staining with the expected pattern. Further validation data can be found under https://www.rockland.com/categories/primary-antibodies/rfp-antibody-200-101-379/
Alexa Fluor 647-conjugated phalloidin was used to stain F-actin in murine cells. We detected specific staining with the expected pattern. Further validation data can be found under https://www. thermofisher.com/order/catalog/product/A22287

Animals and other research organisms

Policy information about studies involving animals; ARRIVE guidelines recommended for reporting animal research, and Sex and Gender in Research

Laboratory animals

C57BL/6 mice were obtained from Charles River Laboratory; The IFN-γR KO mouse (8 weeks) strain (homozygous for the null mutation of the IFN-γR gene) and their WT littemates were used to induce colitis; For lineage tracing of cells derived from AXIN2-expressing cells, Axin2CreERT2/Rosa26-tdTomato mice were generated by breeding Axin2CreErt2 to Rosa26-tdTomato mice. For lineage tracing of cells derived from KRT20-expressing cells, Krt20CreERT2/Rosa26-tdTomato mice were generated by breeding Krt20CreErt2 to Rosa26-tdTomato mice. Mice aged from 6-12 weeks were used in cell extraction. Bmprla fl/fl mice were obtained from the laboratory of Yuji Mishina. To generate conditional KO mice with depletion of Bmpr1a in COL1A1+ cells, we bred Bmpr1a fl/

	fl mice to Col1a1CreERT2 mice. The Col1a2CreERT2/Alk3fl/fl mice (8 weeks) and their WT littermates (8 weeks) were injected with tamoxifen intraperitoneally on three consecutive days to deplete Bmpr1a from Col1a2+ cells. All animals were maintained in autoclaved micro-isolator cages and provided with sterile drinking water and chow ad libitum. The mice were bred at the animal care facility on a 12-h light/12-h dark cycle in a controlled temperature (22 .5 ± 2 .5 °C) and humidity (50 ± 5%) environment.
Wild animals	No wild animals were used in the study.
Reporting on sex	Both sex mice were used in the study.
Field-collected samples	No filed-collected samples were used in the study
Ethics oversight	All procedures involving animals were approved by the institutional, local, and national legal authorities (LaGeSo Berlin, T-CH 0032/20) at the Charite Universitätsmedizin Berlin.

Note that full information on the approval of the study protocol must also be provided in the manuscript.

Plants

Seed stocks	NA
Novel plant genotypes	NA
Authentication	NA
